# Anti-Tumor Activity, *In Vitro* and *In Vivo*, of Some Triphenylphosphinegold(I) Thionucleobases

**DOI:** 10.1155/MBD.1996.63

**Published:** 1996

**Authors:** Lorraine K. Webster, Silvina Rainone, Ernst Horn, Edward R. T. Tiekink

**Affiliations:** 1 Experimental Chemotherapy and Pharmacology Laboratory Peter MacCallum Cancer Institute East Melbourne Victoria 3002 Australia; 2 Department of Chemistry The University of Adelaide Adelaide South Australia 5005 Australia

## Abstract

The [Ph_3_PAu(6-MP)] complex, where 6-MPH is 6-mercaptopurine, is active against the cisplatinresistant cell line, mouse leukaemia L1210/DDP, as is the precursor compound [Ph_3_PAuCl], suggesting that the thiolate is not critical for activity. Against the human cell lines, FaDu (squamous cell carcinoma) and SKOV-3 (ovarian carcinoma), both [Ph_3_PAu(6-MP)] and [Ph_3_PAu(6-TG)], where 6-TGH is 6-thioguanine, were active. [Ph_3_PAu(6-MP)] was active against a murine PC6 plasmacytoma, but not as active as cisplatin.


### ANTI-TUMOR ACTIVITY, *IN VITRO* AND *IN VIVO*, OF SOME TRIPHENYLPHOSPHINEGOLD(I) THIONUCLEOBASES

Lorraine K. Webster,*[1] Silvina Rainone,[1] Ernst Horn[2] and Edward R.T. Tiekink*[2]

[1] Experimental Chemotherapy and Pharmacology Laboratory, Peter MacCallum Cancer Institute, East Melbourne, Victoria 3002, Australia

[2] Department of Chemistry, The University of Adelaide, Adelaide, South Australia 5005, Australia

### Abstract.

The [$Ph_3PAu$(6-MP)] complex, where 6-MPH is 6-mercaptopurine, is active against the cisplatin-resistant cell line, mouse leukaemia L1210/DDP, as is the precursor compound [$Ph_3PAuCl$], suggesting that the thiolate is not critical for activity. Against the human cell lines, FaDu (squamous cell carcinoma) and SKOV-3 (ovarian carcinoma), both [$Ph_3PAu$(6-MP)] and [$Ph_3PAu$(6-TG)], where 6-TGH is 6-thioguanine, were active. [$Ph_3PAu$(6-MP)] was active against a murine PC6 plasmacytoma, but not as active as cisplatin.

### Introduction.

The use of gold compounds in the treatment of rheumatoid arthritis is well documented [1 - 3]. Given the clinical success of such compounds, it is not surprising that gold derivatives have also been examined for their potential anti-tumour activity. Among the most active species investigated was the bis chelated diphosphine gold(I) cation, $[Au(dppe)_2]^+$ , where dppe is $Ph_2PCH_2CH_2PPh_2$ [4, 5]; this complex proved to be a potent cardiovascular toxin [6]. In this context the anti-tumour activity/cytotoxicity of a series of phosphinegold(I) thiolates have been investigated [7, 8] and we have reported the *in vitro* growth inhibitory activity of a series of phosphinegold(I) thionucleobases with the general formula [$R_3PAu$(SR)], where R = Et, Ph or c-hexyl, and SRH = 2-mercaptobenzoic acid, 2-thiouracil, 6-mercaptopurine (6-MPH), or 6-thioguanine (6-TGH) [9]. These latter two thionucleobases are anticancer drugs that are used clinically in the treatment of leukaemia [10]. All of the gold complexes showed excellent growth inhibition activity against the L1210 murine leukaemia *in vitro*. The activity was generally 10 to 100-fold better than for cisplatin and carboplatin, the only other metal-containing anticancer drugs in routine clinical use. The development of resistance to these platinum agents is a significant clinical problem [11], and hence there is a real need for drugs that are able to circumvent platinum resistance. It is also important to determine whether potential new agents have activity against human cancer cell lines, and against tumours growing in mice. Although there was no clear structure-activity relationship in the previous series [9], two triphenylphosphinegold(I) complexes, i.e. [$Ph_3PAu$(6-MP)] and [$Ph_3PAu$(6-TG)], have been chosen for further study, and their activity has been investigated against the cisplatin-resistant L1210 leukaemia, as well as two human carcinoma cell lines, and a murine tumour growing *in vivo*.

### Materials and Methods.

*Materials*

The two compounds [$Ph_3PAu$(6-MP)] and [$Ph_3PAu$(6-TG)] were prepared and purified as described previously [12, 13].

A preliminary crystallographic analysis of [$Ph_3PAu$(6-TG)] [14] shows that the gold atom is coordinated by the S [Au-S 2.30(1) Å] and P [Au-P 2.28(1) Å] atoms and exists in a linear geometry [S-Au-P 175.8(6)°]; a close intramolecular Au...N interaction of 3.23(1) Å is noted. This structure is analogous to that of the previously determined [$Ph_3PAu$(6-MP)] complex [13]. As well as the gold complexes, the free thionucleobases were tested, as was $Ph_3PAuCl$. *Cis*-diamminedichloroplatinum(II) (Institute of Drug Technology, Melbourne, Australia) was used as a control.

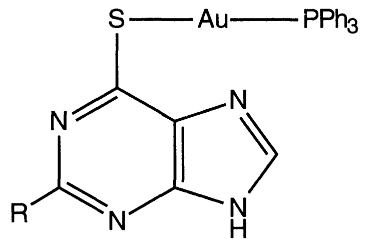

R = H: $[Ph_3PAu(6\text{-}MP)]$

R = $NH_2$: $[Ph_3PAu(6\text{-}TG)]$

*In vitro mouse leukaemia growth inhibition assays*
Sensitive (L1210) or cisplatin-resistant (L1210/DDP) mouse leukaemia cells were grown as suspension cultures in Eagle's minimum essential medium (MEM) plus 1% glutamine and 10% foetal calf serum (FCS, Flow Laboratories). All compounds were dissolved in dimethylsulfoxide (dmso) at 9 concentrations over a 3-log range, with a final maximum dmso concentration in the growth medium of 0.5%. Using 24-well Costar dishes, exponentially growing cells (5 x $10^4$ cells/ml) were incubated with drugs at each concentration in duplicate at 37 °C in a humidified incubator gassed with 5% $CO_2$/95% air for 48 hr. Cell numbers were then counted with a Coulter counter (model ZM). The average number of cells in each duplicate drug-treated culture was then expressed as a percentage of the quadruplicate vehicle-treated control. The $IC_{50}$ value in µM, defined as the concentration of compound required to inhibit 50% of the cell growth after 48 hr of drug exposure, was calculated graphically.

*In vitro human carcinoma growth inhibition assays*
The SKOV-3 human ovarian carcinoma cell line was maintained in α-MEM plus 15% FCS. The FaDu human squamous cell carcinoma of the hypopharynx was maintained in RPMI plus 10% FCS. For the growth inhibition studies, 5 x $10^3$ SKOV-3 or 1 x $10^4$ FaDu exponentially growing cells in 100 ml medium were allowed to adhere in 96-well culture plates for 12 to 16 hr at 37 °C in a humidified incubator (gassed as above). Drugs were dissolved in dmso and diluted in medium to 10 concentrations over a 4-log range, and 100 ml of each drug solution was added to 5 wells. Cells were incubated for a further 72 hr, after which viable cells were measured using the sulforhodamine B (SRB) assay [15] that measures cellular protein content. Briefly, cells were fixed with trichloroacetic acid and stained with SRB. Unbound dye was removed by washing with acetic acid, protein-bound dye was solubilised with Tris base, and the optical density was read at 550 nm using an automatic plate reader. The percentage growth inhibition was calculated as above.

*In vivo mouse anti-tumour activity*
Female Balb/c mice (10-15 weeks) were maintained in controlled atmospheric conditions and fed standard mouse chow and water *ad lib*. The protocol was approved by the Institutional Animal Experimentation and Ethics Committee. The murine PC6 plasmacytoma (obtained from L. Kelland, Institute of Cancer Research, Sutton, UK) was inoculated as 1 mm cubes subcutaneously on the flanks of the mice, and approximately 20 days later, mice with tumours were randomised into groups of 5 to 10 animals, which received either nothing (no-drug control), or an intraperitoneal injection of dmso at 2.5 ml/kg (vehicle control), cisplatin in saline at 8 mg/kg (positive control), or the test drugs at the maximum tolerated dose at 2.5 ml/kg in dmso. Eight to ten days later, mice were sacrificed and the tumours were dissected and weighed. The compounds tested against this tumour were $[Ph_3PAuCl]$, [6-MPH], $[Ph_3PAu(6\text{-}MP)]$ and cisplatin.

### Results.

*In vitro growth inhibition*
The results for the *in vitro* growth inhibition studies are summarised in Table 1. The gold complexes $[Ph_3PAuCl]$ and $[Ph_3PAu(6\text{-}MP)]$ were both active against the cisplatin-resistant L1210, whereas [6-MPH] lost its activity. The gold complexes were both active against the human cell lines, whereas the free thionucleobases were not. The presence of the thionucleobase within the gold complex did not add to the activity against the SKOV-3 cells.

**Table 1.** *In vitro* growth inhibition activity.

| Compound | L1210 | L1210/DDP | FaDu | SKOV-3 |
|---|---|---|---|---|
| [$Ph_3PAuCl$] | 0.30 | 0.14 | n.d. | 0.13 |
| [6-MPH] | 0.310* | 3.3 | >5 | 19.5 |
| [$Ph_3PAu$(6-MP)] | 0.083* | 0.05 | 0.25 | 0.36 |
| [6-TGH] | 0.096* | n.d. | >2 | 9.1 |
| [$Ph_3PAu$(6-TG)] | 0.052* | n.d. | 0.39 | 0.63 |
| Cisplatin | 0.6 | 6.7 | 6.1 | 3.1 |

Values are the $IC_{50}$ in μM of 2 to 5 experiments.
n.d. = not done.
* these results have been published previously [9]

*In vivo anti-tumour activity*
[6-MPH] at 50 mg/kg and [$Ph_3PAuCl$] at 25 mg/kg were not effective in reducing the size of the PC6 tumours. However, treatment with a single dose of [$Ph_3PAu$(6-MP)] at 25 mg/kg led to a 60% reduction in tumour size. Although the decrease seen with [$Ph_3PAu$(6-MP)] was statistically significant (P=0.006, one way analysis of variance), cisplatin was able to cure all of the mice.

### Discussion.

The [$Ph_3PAu$(6-MP)] complex showed impressive activity against the cisplatin-resistant cells. There are several reported mechanisms of platinum resistance in this L1210/DDP cell line, including reduced drug uptake, increased intracellular glutathione levels, and increased DNA repair following platination [16 - 18]. It is not clear which mechanism is most important in the present case, but it is apparent that the addition of the thionucleobase to the gold complex is not necessary to overcome the cisplatin resistance, as [$Ph_3PAuCl$] was also found to have some activity, although not as pronounced. The complexes [$Ph_3PAu$(6-MP)] and [$Ph_3PAu$(6-TG)] showed equivalent activity against both of the human cell lines, whereas the free thionucleobases were inactive. Again, addition of the thionucleobase to the complexes did not enhance this activity.

Despite the encouraging *in vitro* growth inhibition activity, the *in vivo* results were disappointing. Activity at least as good as cisplatin against animal tumours would ensure a positive response towards further development. Complexes of the general structure $R_3PAuSR$, such as Auranofin [2,3,4,6-tetra-*O*-acetyl-1-thio-β-D-glucopyranosato-*S*-triethylphosphinegold(I)], have been shown to have cytotoxic activity *in vitro*, but only limited *in vivo* anti-tumour activity [7, 19]. Other gold(I) coordination complexes have also been screened, however, in general their *in vivo* activity is low [20]. This lack of *in vivo* activity may be attributed to the ability of linear gold(I) complexes to undergo rapid ligand-exchange reactions with endogenous thiols in the plasma [21], thus decreasing their availability for cytotoxic activity. For example, decreased *in vitro* activity has been demonstrated for both Auranofin [7] and triphenylphosphinegold(I) 8-thiotheophyllinate [8] when increasing concentrations of albumin were present in the growth medium. Thus, further *in vitro* studies with these triphenylphosphinegold(I) thionucleobases will be necessary to assess their reactivity with biological solutions. In addition, it is important to determine the mechanism of action of these compounds in order to guide further development and testing.

### Acknowledgments.

The Australian Research Council is thanked for support of this work.

**References.**

1. Lewis, A.J.; Walz, D.T. *Prog. Med. Chem.*, 1982, **19**, 1.
2. Sutton, B.M. *Gold Bull.*, 1986, **19**, 15.
3. Parish, R.V. *Interdisciplinary Sci. Rev.*, 1992, **17**, 221.
4. Berners-Price, S.J.; Mirabelli, C.K.; Johnson, R.K.; Mattern, M.R.; McCabe, F.L.; Faucette, L.F.; Sung, C.-M.; Mong, S.-M.; Sadler, P.J.; Crooke, S.T. *Cancer Res.*, 1986, **46**, 5486.
5. Berners-Price, S.J.; Girard, G.R.; Hill, D.T.; Sutton, B.M.; Jarrett, P.S.; Faucette, L.F.; Johnson, R.K.; Mirabelli, C.K.; Sadler, P.J. *J.Med. Chem.*, 1990, **33**, 1386.
6. Rush, G.F.; Alberts, D.W.; Meunier, P.; Leffler, K.; Smith, P.F. *Toxiocologist*, 1987, **7**, 59.
7. Mirabelli, C.K.; Johnson, R.K.; Sung, C.-M.; Faucette, L.F.; Muirhead, K.; Crooke, S.T.; *Cancer Res.*, 1985, **45**, 32.
8. Arizti, M.P.; Garcia-Orad, A.; Sommer, F.; Silvestro, L.; Massiot, P.; Chevallier, P.; Gutiérrez-Zorrilla, J.M.; Colacio, E.; Martinez de Pancorbo, M.; Tapiero, H. *Anticancer Res.*, 1991,**11**, 625.
9. Tiekink, E.R.T.; Cookson, P.D.; Linahan, B.M.; Webster, L.K. *Metal Based Drugs*, 1994, **1**, 299.
10. McCormack, J.J.; Johns, D.G. in *Cancer Chemotherapy: Principles and Practice*, Chabner, B.A.; Collins, J.M. (Eds) Lippincott Company, Philadelphia, 1990, Chapter 9.
11. Marshall, J.L.; Andrews P.A. *Hematol/Oncol Clinics N Amer.*, 1995, **9**, 415.
12. Harker, C.S.W.; Tiekink, E.R.T.; Whitehouse, M.W. *Inorg. Chim. Acta*, 1991, **181**, 23.
13. Cookson, P.D.; Tiekink, E.R.T.; Whitehouse, M.W. *Aust. J. Chem.*, 1994, **47**, 577.
14. Crystal data for [$Ph_3PAu$(6-TG)]: hexagonal space group $P6_1$, $a$ = 22.291(4) Å, $c$ = 11.094(5) Å, $V$ = 4773(1) $Å^3$, $Z$ = 6, $R$ = 0.059 for 792 data.
15. Skehan, P.; Storeng, R.; Scudiero, D.; Monks, A.; McMahon, J.; Vistica, D.; Warren, J.; Bokesch, H.; Kennedy, S.; Boyd, M. *J. Natl Cancer Inst.*, 1990, **82**, 1107.
16. Kraker, A.J.; Moore, C.W. *Cancer Res* ., 1988, **48**, 9.
17. Tashiro, T.; Sato, Y. *Jpn J. Cancer Res.*, 1992, **83**, 219.
18. Richon, V.M.; Schults, N.; Eastman, A. *Cancer Res.* , 1987, **47**, 2056.
19. Simon, T.M.; Kunishima, D.H.; Vibert, G.J.; Lorber, A. *Cancer Res* ., 1981, **41**, 94.
20. Mirabelli, C.K.; Johnson, R.K.; Hill, D.T.; Faucette, L.F.; Girard, G.R.; Kuo, G.Y.; Sung, C.-M.; Crooke, S.T. *J. Med. Chem.*, 1986, **29**, 218.
21. Shaw, C.F. Gold. In: Metal Compounds in Cancer Therapy. Fricker, S.P. (Ed.). Chapman & Hall, London, 1994, pp 46 - 64.

**Received: January 12, 1996 - Accepted: January 19, 1996 - Received in revised camera-ready format: January 29, 1996**

